# Usability and Acceptance of Wearable Biosensors in Forensic Psychiatry: Cross-sectional Questionnaire Study

**DOI:** 10.2196/18096

**Published:** 2021-05-10

**Authors:** Pieter Christiaan de Looff, Henk Nijman, Robert Didden, Matthijs L Noordzij

**Affiliations:** 1 Behavioural Science Institute Radboud University Nijmegen Netherlands; 2 De Borg Den Dolder Netherlands; 3 Fivoor Science and Treatment Innovation Den Dolder Netherlands; 4 Trajectum Zwolle Netherlands; 5 Department of Psychology, Health and Technology University of Twente Enschede Netherlands

**Keywords:** forensic psychiatry, wearable biosensors, intellectual disabilities, usability, acceptance, continuous use, emotion regulation, behavior regulation

## Abstract

**Background:**

The use of wearable biosensor devices for monitoring and coaching in forensic psychiatric settings yields high expectations for improved self-regulation of emotions and behavior in clients and staff members. More so, if clients have mild intellectual disabilities (IQ 50-85), they might benefit from these biosensors as they are easy to use in everyday life, which ensures that clients can practice with the devices in multiple stress and arousal-inducing situations. However, research on (continuous) use and acceptance of biosensors in forensic psychiatry for clients with mild intellectual disabilities and their caretakers is scarce. Although wearable biosensors show promise for health care, recent research showed that the acceptance and continuous use of wearable devices in consumers is not as was anticipated, probably due to low expectations.

**Objective:**

The main goal of this study was to investigate the associations between and determinants of the expectation of usability, the actual experienced usability, and the intention for continuous use of biosensors.

**Methods:**

A total of 77 participants (31 forensic clients with mild intellectual disabilities and 46 forensic staff members) participated in a 1-week trial. Preceding the study, we selected 4 devices thought to benefit the participants in domains of self-regulation, physical health, or sleep. Qualitative and quantitative questionnaires were used that explored the determinants of usability, acceptance, and continuous use of biosensors. Questionnaires consisted of the System Usability Scale, the Technology Acceptance Model questionnaire, and the extended expectation confirmation model questionnaire.

**Results:**

Only the experienced usability of the devices was associated with intended continuous use. Forensic clients scored higher on acceptance and intention for continuous use than staff members. Moderate associations were found between usability with acceptance and continuous use. Staff members showed stronger associations between usability and acceptance (*r*=.80, *P*<.001) and usability and continuous use (*r*=.79, *P*<.001) than clients, who showed more moderate correlations between usability and acceptance (*r*=.46, *P*=.01) and usability and continuous use (*r*=.52, *P*=.003). The qualitative questionnaires in general indicated that the devices were easy to use and gave clear information.

**Conclusions:**

Contrary to expectations, it was the actual perceived usability of wearing a biosensor that was associated with continuous use and to a much lesser extent the expectancy of usability. Clients scored higher on acceptance and intention for continuous use, but associations between usability and both acceptance and continuous use were markedly stronger in staff members. This study provides clear directions on how to further investigate these associations. For example, whether this is a true effect or due to a social desirability bias in the client group must be investigated. Clients with mild intellectual disabilities might benefit from the ease of use of these devices and their continuing monitoring and coaching apps. For these clients, it is especially important to develop easy-to-use biosensors with a minimum requirement on cognitive capacity to increase usability, acceptance, and continuous use.

## Introduction

The use of wearable biosensor devices for monitoring and coaching in forensic psychiatric settings for people with intellectual disabilities and their caretakers yields high expectations for improved self-regulation of emotions and behavior. This is based on the expectation that wearable biosensor devices can be used to detect changing levels of emotional states [1] and behavior in both clients and staff members [2-5]. The devices typically collect body measurements such as heart rate, blood pressure, movement, and breathing [6,7]. It is assumed that this information can be used to quantify health, physiological stress, and well-being [8]. Users can, for instance, visualize their data in smartphone apps to track their health and stress levels or receive real-time information on their heart rate coupled to personalized, prespecified interventions [2]. This information is thought to enhance health and well-being and alleviate stress [2,6,8]. Besides the detection and prediction of emotional states and behavior, wearable biosensors may be used to promote a healthy lifestyle, especially because of their real-time data monitoring capabilities [3,9]. For example, they can be used to monitor sleep [10], nutrition, movement, cardiac disease, blood sugar [3], or epilepsy [8]. In these cases, wearable technology has the potential to lower medical costs, improve health-related behavior of users, and reduce physician time [3]. In addition, it may result in opportunities to gather accurate real-time data that will allow for the diagnosis, prevention, and treatment of various chronic diseases in a more economical manner [11-13]. This makes the potential economic benefits of these biosensors immense; health and fitness biosensors are targeted at improving healthy behavior, which will likely have a significant impact on health care costs [11]. Although some results from recent research in forensic psychiatry are promising [4,14], the (continuous) use of biosensors in everyday life, particularly in forensic psychiatry, is still in nascent stages [15].

A potentially complicating factor in the use of these biosensors are the mild intellectual disabilities and borderline intellectual functioning (MID-BIF; IQ 50-85) of the user. Clients with MID-BIF might not benefit from cognitive behavioral therapies (eg, anger management) like people with average intelligence [16] due to the complexity of such therapies. The use of biosensors might be an easier method by which to teach people about arousal-inducing events and possible self-regulation strategies. Moreover, the ambulatory nature of the devices ensures that clients can practice with the devices in multiple stress-inducing situations occurring in real life.

Although wearable biosensors show promise for health care, research into the use and acceptance of wearable biosensors is almost absent in forensic psychiatry, let alone in clients with MID-BIF. Recent consumer research, however, showed that the acceptance of wearable devices in consumers is not as was anticipated [3], and, more importantly, the long-term continuous use (presumably following acceptance) of these devices seems low [17]. In addition, there is a need for more longitudinal research to systematically study the trends in wearable use over time. Pal et al [12] recently identified factors that might contribute to the low frequency of continuous use of wearable devices. According to these authors, there is a gap between the expectations of usability that people have before using, for example, a smartwatch and the factors that would lead to continuous use by experiences with the device (expectation–experience–continuous use). In this study, we will investigate whether it is the expectation of using the biosensor or the actual experience itself that contributes to continuous use. Examples of factors associated with usability, acceptance, and continuous use of wearable biosensor devices include the accuracy of the information from the devices, reliability and validity of the information, comfort of the devices, feedback provided, and how it is provided [3]. For instance, if a wearable biosensor device signals that a client is stressed while the client is clearly at ease, this will likely reduce the willingness to use the biosensor, as the accuracy, reliability, and validity of the information is clearly erroneous in this example.

To increase the usability, acceptance, and continuous use of biosensors, user preferences, needs, and wishes, especially for people with MID-BIF, must be known. In addition, it is necessary to determine the goals of the user and the tasks for which the biosensors will be used. Finally, the functions of user interfaces and biosensor devices should be evaluated to make them more attractive, desirable, and efficient for the users by integrating the outcomes of the evaluation [17,18]. Kim and Shin [15] argued that additional antecedents of biosensor adoption and the role of control variables should be further investigated to increase the (continuous) use and acceptance in diverse international samples. In addition, Kalantari [11] argued for more diverse samples and heterogeneous user groups. Kalantari [11] also notes that qualitative research is lacking in the field of acceptance and adoption research.

Research on the use of biosensors for clients with MID-BIF is scarce while the potential benefits might be significant. Therefore, we investigated the use of everyday wearable biosensors to establish what would lead to their (continuous) use and acceptance. Biosensor information could potentially benefit not only clients but staff members as well. To this end, qualitative and quantitative questionnaires were used to explore the psychological (preferences, needs, wishes, and goals) and functional (tasks and functions) determinants of usability, acceptance, and continuous use of biosensors in both staff members and clients. The main goal of this study was to investigate the expectation–experience–continuous use connection to see whether there is a gap between expectations of usability and the actual experience that will preferably lead to continuous use and whether there are differences between clients and staff members. In addition, we investigated the key determinants involved in the usability, acceptance, and continuous use of biosensors using validated questionnaires. The following research questions were formulated:

- Are there differences between clients and staff members in expectations of usability and the actual experienced usability that will lead to continuous use of biosensors (expectation–experience–continuous use)?
- Which key determinants contribute to the usability, acceptance, and continuous use of biosensors in forensic psychiatry for clients with MID-BIF and staff members?

## Methods

### Participants and Setting

The participants for this small-scale study consisted of two groups of users: clients with MID-BIF who are residents of forensic psychiatric living units and staff members who work as nurses or sociotherapists on these forensic psychiatric living units. Clients are often referred to the units as a result of aggressive and violent behavior and are at an increased risk for severe behavior problems, offending behavior, and recidivism [19]. During their admission, these clients are encouraged to participate in treatments aimed at decreasing the risk for recidivism. The staff members who work with these clients often work irregular shifts and are at an increased risk for work-related stress and burnout symptoms [4].

### Materials

After multiple sessions with a user group consisting of staff members and clients, we selected 4 devices that were thought to benefit the participants in domains of self-regulation, physical health, or sleep. All 4 devices used in this study are US Food and Drug Administration and CE approved and can be bought in regular stores (commercially available).

The Spire Stone (Spire Health) is a wearable device in the form of a stone that can be attached to a belt (men) or bra (woman). It measures the contraction of the torso to indicate the rate of breathing. The device comes with an app and classifies the respiration rate as calm, focused, or tense (see Holt et al [20], for instance). Moreover, it measures the amount of activity and sedentary behavior. The app provides users with daily overviews or direct feedback on breathing rate.

The Charge 3 (Fitbit Inc) is a physical activity tracker with a heart rate monitor that provides users with real-time feedback on heart rate and physical activity. In addition, it can provide users with information on sleep and exercise. The app provides users with detailed information on stress, sleep, and activity (see Schrager et al [21], for instance).

The vívosmart 4 (Garmin Ltd) is a physical activity tracker with a heart rate monitor that provides users with real-time feedback on energy expenditure, stress indications based on heart rate, and sleep quality assessment. The app provides users with detailed information on sleep, stress, and energy expenditure [22].

The TicWatch E (Mobvoi Inc) is a smartwatch running WearOS with a heart rate sensor. It can be used as a biofeedback device when running the Sense-It app [2], an ambulatory e-coaching app that provides users with information on deviations in their regular heart rate. It is aimed at supporting users to better understand and recognize changes in their arousal levels.

### Questionnaires

To assess determinants of usability, we evaluated user satisfaction with the wearable biosensors [3] with the System Usability Scale (SUS), a short 10-item questionnaire. To assess determinants of acceptance, we used the Technology Acceptance Model (TAM) questionnaire, one of the most frequently used theoretical frameworks for the acceptance of new technology [15]. To assess determinants of continuous use, we used a questionnaire devised by Pal et al [12] based on an extended expectation confirmation model (EECM) that consists of 10 prime factors associated with the continuous use of smartwatches.

As the questionnaires were not available in Dutch, they were translated by 3 researchers and 8 staff members who work in forensic psychiatric settings with MID-BIF clients. The questionnaires were then back-translated by native English speakers. As the formulation of the questions was deemed too complex for the MID-BIF clients, an easier version of all 3 questionnaires was constructed for MID-BIF clients consisting of fewer, more easily formulated questions. The choice of which questions to select for the short version for clients was made by the researchers and staff members based on two key principles: the question should easily be understood by the MID-BIF client and represent the implied construct to be measured.

### Usability

The SUS is a 10-item questionnaire with good reliability (.85) [23,24] that can be used to assess determinants of subjective usability. It is widely used to assess the usability of different types of technology such as medical devices, software, and websites. It has a well-established standard reference [3] and is quick to administer. The SUS is scored on a 5-point Likert scale ranging from strongly disagree to strongly agree. The total score is obtained by adding the positively worded items minus 1. For negatively worded items, the score is subtracted from 5. The scores are then summed and multiplied by 2.5 to obtain a value between 0 and 100. Missing values can be assigned a neutral value of 3 in accordance with recommendations [25].

To answer the first research question on the expectation–experience–continuous use connection, the questionnaire was administered twice. The SUS questionnaire was administered preceding the study to measure the expectation of the participants. Following 1 week, the SUS was administered to measure the actual experience with the biosensors.

### Acceptance

The TAM questionnaire [26] is one of the most frequently used questionnaires for the acceptance of new technology [27]. The model uses a generalized theoretical framework of technology. We used a recently developed version of this questionnaire [15] specifically aimed at smartwatches that distinguishes between 10 key determinants of smartwatches: perceptions of and attitudes toward technology, affective quality, relative advantage, mobility, availability, perceived ease of use, intention to use, perceived usefulness, subcultural appeal, and cost. The TAM is scored on a 7-point Likert scale ranging from strongly disagree to strongly agree. The questionnaire consists of 36 questions, and all scales have reliabilities well over .70.

### Continuous Use

Pal et al [12] proposed an EECM consisting of 10 prime factors associated with the continuous use of smartwatches: hedonic motivation, self-socio motivation, perceived privacy, perceived comfort, battery-life concern, perceived accuracy with functional limitations, perceived usefulness, confirmation, satisfaction, and continuous use. The EECM is scored on a 7-point Likert scale ranging from strongly disagree to strongly agree. The calculation of the total EECM score follows a similar logic as the SUS calculation. The positively phrased EECM questions were summed with the value of the score minus 1. The negatively phrased EECM questions were scored with a value of 7 minus the score (note that we did not multiply the sum as is common with the SUS). This was thought to give an adequate indication of continuous use intention. The EECM questionnaire consists of 32 questions, and all scales have reliabilities well over .70.

### Short Questionnaires

The full TAM and EECM questionnaires would be too much of a burden for the clients with MID-BIF. Therefore, we selected one question from each factor on the TAM and EECM for the clients to answer. The staff members completed the full version of the questionnaire. For ease of reporting and interpretation for both clients and staff members, the results reported in this paper consist of the SUS, the short version of the TAM, and the short version of the EECM (Multimedia Appendix 1). All short questionnaires had an overall Cronbach 𝛼>.80 in this study.

### Qualitative Questionnaires

The qualitative questionnaires consisted of an individually administered semistructured interview based on the quantitative questionnaires. Participants were asked to elaborate on thoughts they had on the aspects of usability, acceptance, and continuous use. Additional determinants of usability, acceptance, and continuous use were derived from the semistructured interview in order to further explore the second research question.

### Procedure

The research was conducted from May to August 2019. Recruitment of the participants was done at the sites of the living units. Participants were invited to participate and informed about the aim of the study through posters, flyers, and email. After a participant agreed to participate in the study, wearable biosensors were given to the participants with instructions on how to use them. If a user did not own a phone to connect to the app, a P smart (Huawei Device Co Ltd) was provided to the participant (although some participants were not allowed to use a phone due to the nature of their sentence and only used the app in the presence of their caretakers). One of four commercially available devices was randomly assigned to the participant. Before wearing the device, they completed the SUS questionnaire to assess their expectations of usability. The research coordinator completed the SUS questionnaire with the participant if necessary. Sheehan and Hassiotis [28] identify several reasons why people with intellectual disabilities might experience barriers to use technology including cognitive limitations, physical and sensory impairment, and a lack of training and support. For this reason, the research coordinators assisted the clients in explaining how the devices work and completing the questionnaires.

The participants were given time to get familiar with the biosensors. The research coordinator functioned as a contact person in case the participant had any technical problems [2]. After the participant wore the device for 1 week, they completed the questionnaires on usability, acceptance, and continuous use of the wearable biosensors (which thus also resulted in a measure of experience with the device) again. In-depth qualitative interviews were conducted with 29% (22/77) of participants, 14 of whom were staff members.

### Statistical Analysis

Descriptive statistics were used to describe the devices that were worn and the age, education, and gender of participants. A 2-way mixed analysis of variance (within: pre-post and between: client-staff) was used to test the main outcome on the expectation of usability with the actual experience. The SUS scores were calculated both preassessment and postassessment for each participant to determine their correlation with continuous use to answer the first research question. To test whether there was an association between the expected and experienced usability with (the intended) continuous use of the EECM questionnaire, we used Spearman correlations to answer the second research question, as the scores on the SUS were not normally distributed. To determine the key determinants that contribute to the usability, acceptance, and continuous use of biosensors, the proportion and number of responses for all questionnaires was computed for clients and staff members.

Further exploratory statistical analyses consisted of a 2-way analysis of covariance (ANCOVA) to test which demographic factors were associated with the judgments of the participants concerning the usability, acceptance, and continuous use of biosensors. Last, an analysis was performed on the qualitative questions of usability, acceptance, and continuous use regarding word frequency. All analyses were done in R (version 3.6.1, R Foundation for Statistical Computing) software [29].

## Results

### Participants and Setting

To investigate whether there were differences between clients and staff members in expectations of usability and the actual experienced usability that will lead to continuous use, 77 participants were included (31 clients and 46 staff members), with an age range varying from 18 to 63 (mean 34.9 [SD 10.8]) years. Participants were included from 4 mental health institutions in the Netherlands that provide forensic care for clients with MID-BIF.

### Materials

Participants wore a Charge 3 (31/77), vívosmart 4 (21/77), Spire Stone (14/77), or TicWatch E (11/77; Table 1). The difference between groups in terms of number of worn devices was mainly due to difficulty in including participants at different locations as the sites were randomly assigned two devices each. More staff members than clients were included. Some clients and staff members did not want to answer questions regarding their gender (1/77) or education level (3/77) and were therefore set to missing.

**Table 1:** Descriptive statistics.

Participant			Client (n=31), n (%)		Staff (n=46), n (%)
**Device**					
	Charge 3	16 (52)		15 (33)	
	vívosmart 4	7 (23)		14 (30)	
	Spire Stone	3 (10)		11 (24)	
	TicWatch E	5 (16)		6 (13)	
**Gender^a^**					
	Male	20 (65)		21 (46)	
	Female	10 (32)		25 (54)	
**Education^a^**					
	Primary	16 (52)		0 (0)	
	Secondary	13 (42)		17 (37)	
	Higher	0 (0)		28 (61)	

**^a^**Some participants were reluctant to answer questions on gender and education.

A small proportion of clients with MID-BIF were not allowed to use a mobile phone (6/77). They were therefore unable to answer questions regarding the use of the biosensor in combination with the app. The missing values were therefore imputed with the “don’t know or neutral” categories of the questionnaires. Table 2 shows the total SUS scores at the start and end of the study period for each device. The SUS scores for most devices increased comparing prescores and postscores except for the Spire Stone (both clients and staff) and TicWatch E (only staff decreased).

**Table 2:** Descriptive statistics of System Usability Scale scores.

Group	Product Start	n	Start	SD	End	SD end	Min start	Max start	Min end	Max end
Client	Charge 3	16	56.88	20.01	60.31	18.66	15.00	90.00	15.00	82.50
Client	vívosmart 4	7	66.43	16.00	67.86	17.82	45.00	87.50	47.50	95.00
Client	Spire Stone	3	64.17	10.10	63.33	14.65	55.00	75.00	52.50	80.00
Client	TicWatch E	5	51.50	8.59	57.50	27.33	37.50	60.00	20.00	82.50
Staff	Charge 3	15	69.17	10.42	75.50	12.00	55.00	87.50	55.00	92.50
Staff	vívosmart 4	14	74.11	5.24	76.25	8.31	65.00	82.50	65.00	92.50
Staff	Spire Stone	11	64.77	6.56	59.77	20.05	55.00	77.50	12.50	75.00
Staff	TicWatch E	6	70.83	16.93	44.58	13.73	50.00	97.50	30.00	70.00

The mean for the clients increased over time while the mean for the staff decreased (Table 3). Paired samples *t* tests showed that these differences were nonsignificant for both clients (*t*=–0.647, *P*=.52) and staff members (*t*=0.645, *P*=.52). The discrepancy between the experienced (posttest) and expected (pretest) usability seems to have been caused by the Spire Stone and TicWatch E (see Table 2). There was no interaction effect of expected versus experienced for group (F_1,75_=1.82, *P*=.18).

The correlation between the expected usability and the EECM was .18 (*P*=.12). The correlation between the experienced usability and the EECM was .54 (*P*<.001), which indicates a higher relevance for continuous use after participants wore the device.

**Table 3:** Descriptive statistics of System Usability Scale scores per group.

Group	Time	Variable	n	Mean (SD)
Client	Start	Score	31	58.87 (17.19)
Staff	Start	Score	46	69.84 (9.77)
Client	End	Score	31	61.85 (19.09)
Staff	End	Score	46	67.94 (17.45)

### Usability

**Figure 1:**
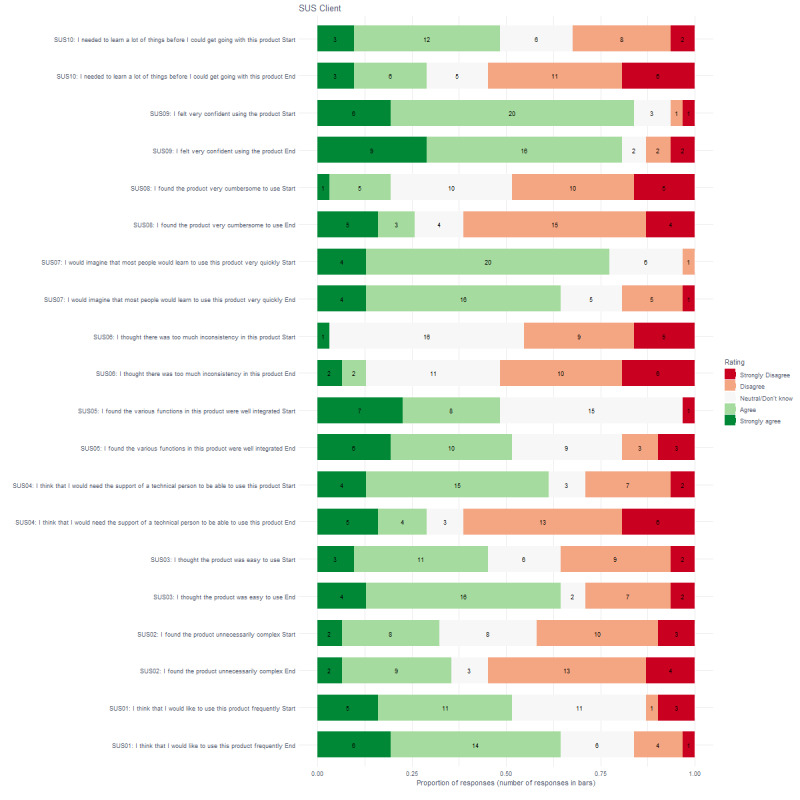
Proportion and number of responses for clients on the System Usability Scale questionnaire.

**Figure 2:**
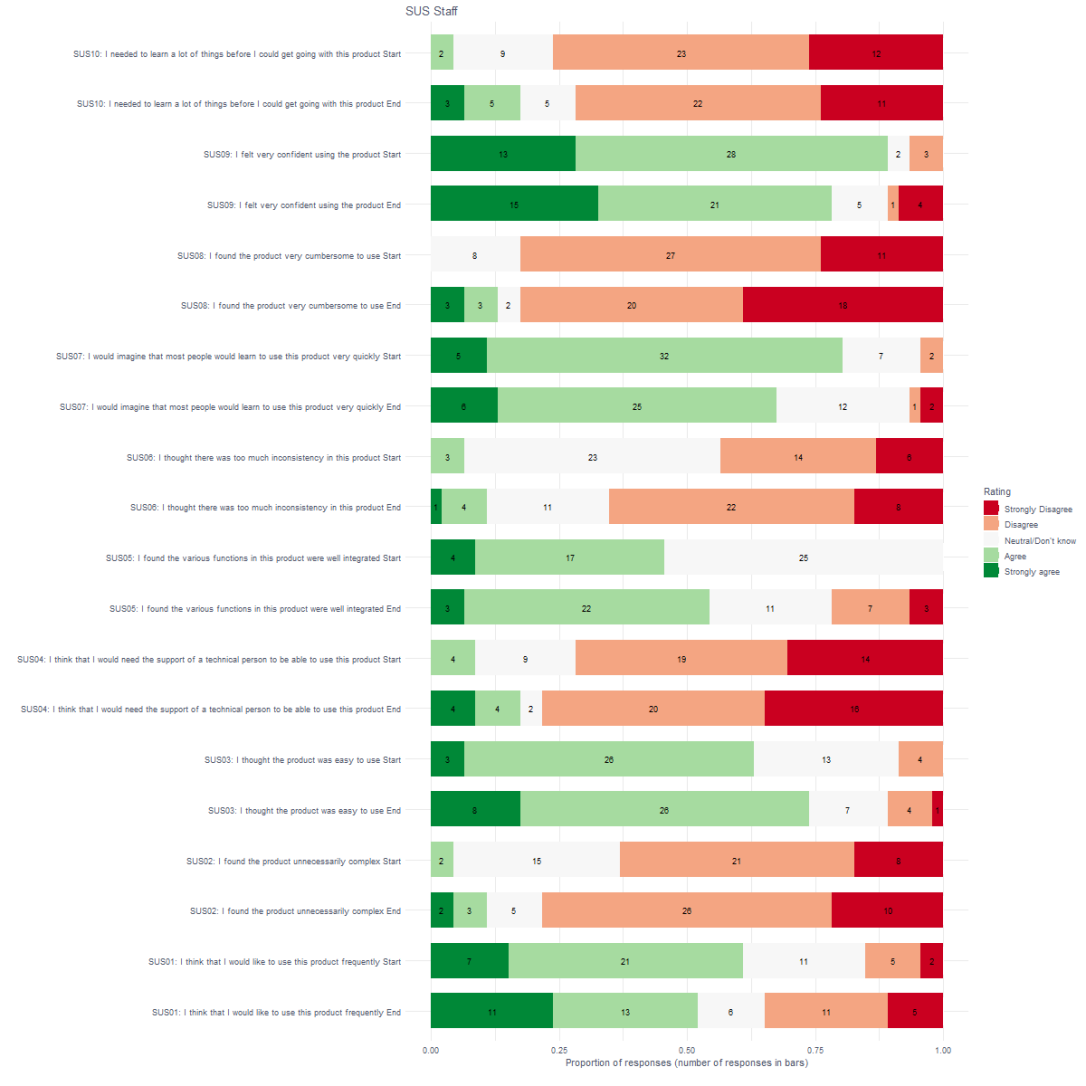
Proportion and number of responses for staff on the System Usability Scale questionnaire.

As shown in Figure 1 and Figure 2, at the start and end of the study, more than 75% of clients (start 26/31; end 25/31) and staff members (start 41/46; end 36/46) indicated that they were confident to use the biosensors. More than 50% of clients (start 24/31; end 20/31) and staff members (start 37/46; end 31/46) also indicated that they could imagine that most people would learn to use the biosensor very quickly, and both clients (start 16/31; end 20/31) and staff members (start 28/46; end 24/46) would like to use it frequently. At the start of the study, 61% (19/31) of clients indicated they needed help from a technical person, in comparison to 29% (9/31) at the end of the study. More than 50% of staff members (start 29/46; end 34/46) thought the biosensor was easy to use, and by the end of the study, more than 50% of clients also agreed with that (20/31). More than 50% of staff members (start 29/46; end 36/46) did not agree that the biosensors were unnecessarily complex, did not agree that they would need the support of a technical person (start 33/46; end 36/46), did not agree that the biosensors were very cumbersome to use (start 38/46; end 38/46), and did not agree that they needed to learn many things before they could get going with the biosensors (start 35/46; end 33/46).

### Acceptance

**Figure 3:**
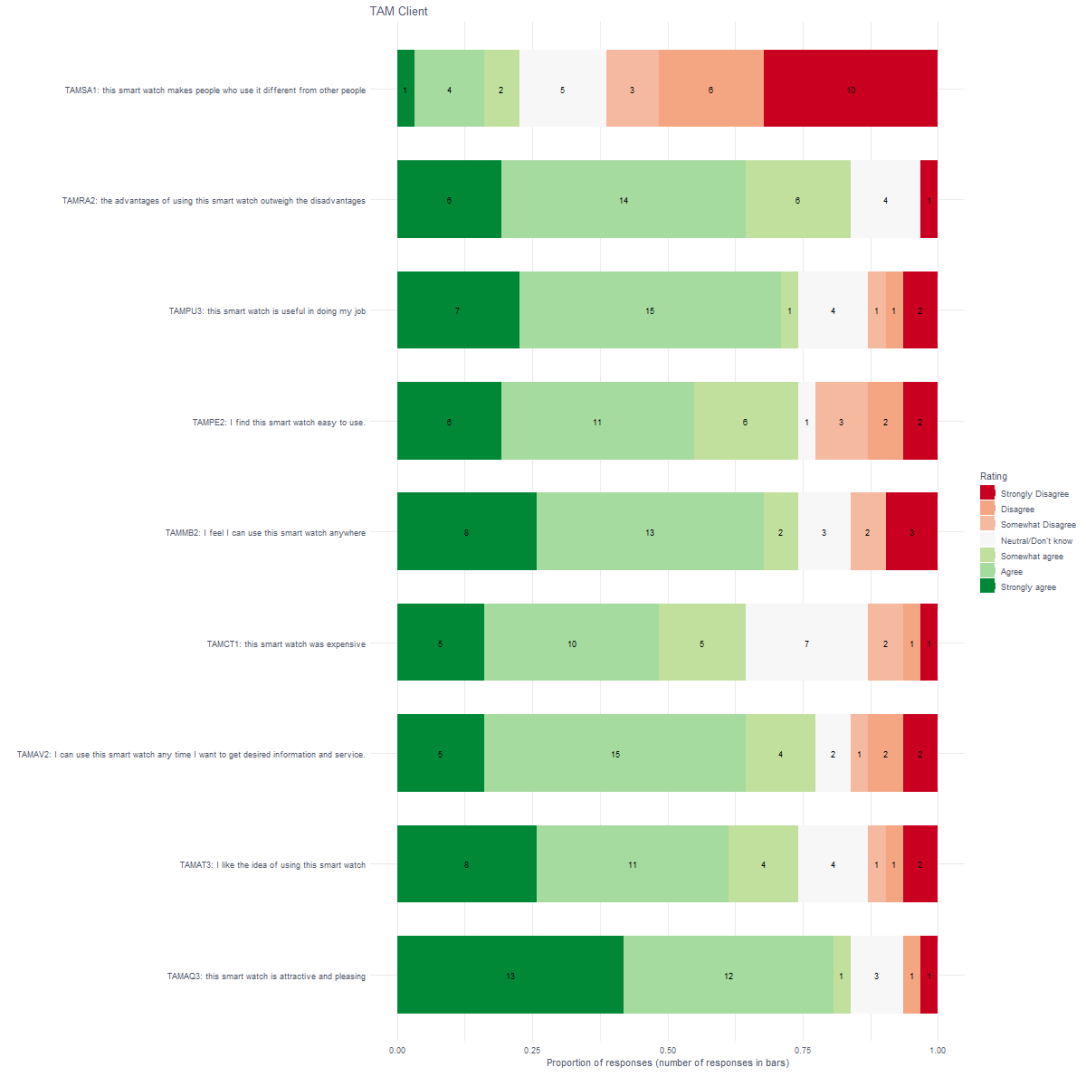
Proportion and number of responses for clients on the Technology Acceptance Model questionnaire.

**Figure 4:**
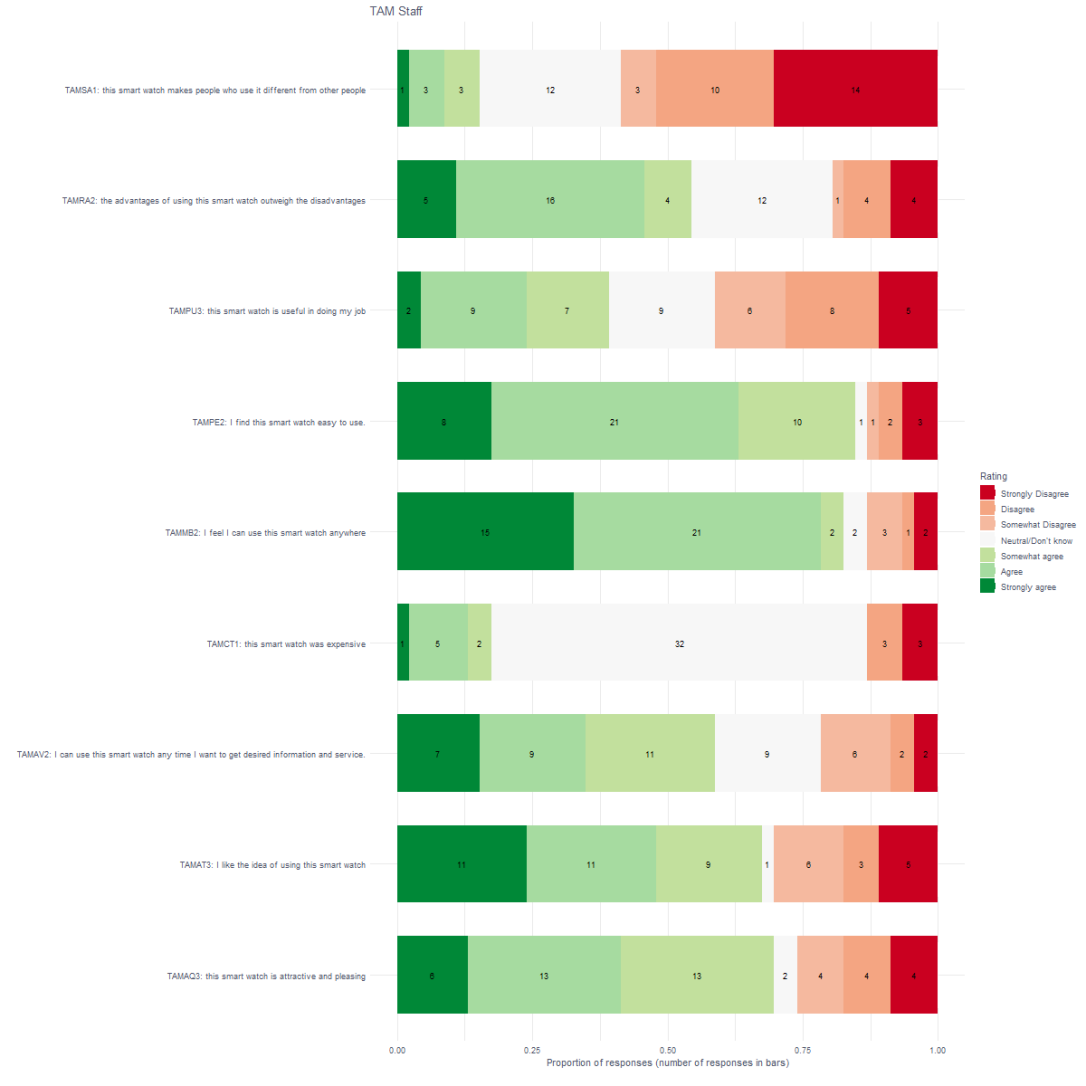
Proportion and number of responses for staff on the Technology Acceptance Model questionnaire.

Figure 3 shows that more than 75% of clients thought that the smart watch was attractive and pleasing (26/31), they could use the device to get the desired information and service (24/31), and the advantages outweighed the disadvantages (26/31). More than 50% of clients liked the idea of using the smartwatch (23/31), thought it was expensive (20/31), felt they could use the smartwatch anywhere (23/31), was easy to use (23/31), and was useful for doing their job (26/31). Figure 4 shows that staff members had similar patterns except that a small number of staff members thought that the smartwatch was expensive (8/46) or that it was useful for doing their job (18/46).

### Continuous Use

**Figure 5:**
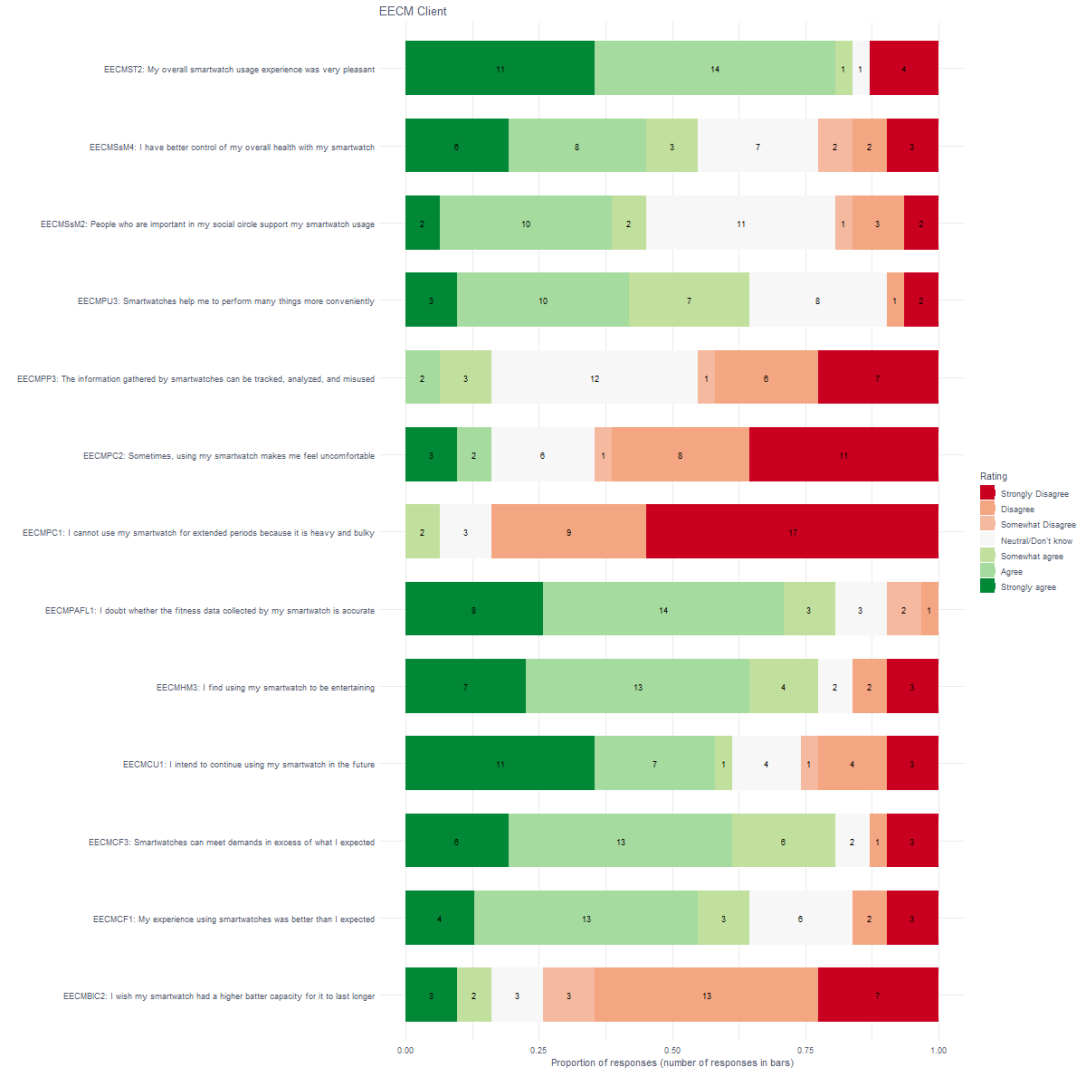
Proportion of responses and number of responses for clients on the extended expectation confirmation model.

**Figure 6:**
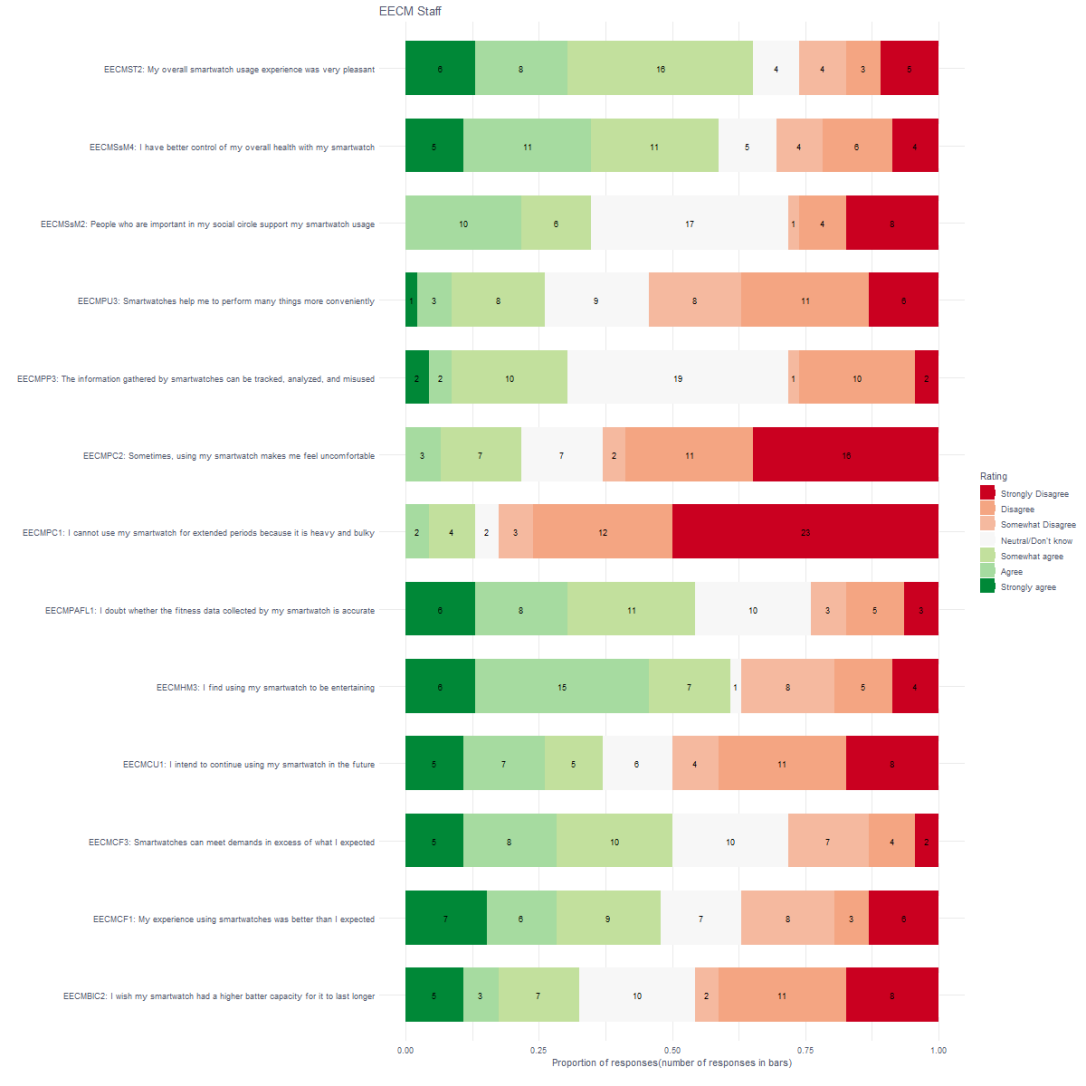
Proportion of responses and number of responses for staff on the extended expectation confirmation model.

Figure 5 shows that more than 75% (26/31) of clients agreed to some extent that it was a pleasant experience, that it was entertaining (24/31), that the biosensors met demands in excess of what they expected (25/31), and that they doubted whether the fitness data were accurate (25/31). In addition, more than 75% (26/31) of clients disagreed that the biosensors were heavy and large, and that the devices needed a larger battery capacity (25/31). More than 50% of clients agreed that the experience was better than expected (20/31), that they intend to use the device in the future (19/31), that the device helps them perform many things more conveniently (20/31), and that they have better control over their health (17/31) and did not agree that using the smart watch makes them feel uncomfortable (20/31). Figure 6 shows that the outcome for staff members was slightly different, although the patterns look similar at first glance. More than 75% (38/46) of staff members disagreed that the biosensors were heavy and large. More than 50% of staff members found the use entertaining (27/46), doubted that the fitness data were accurate (25/46), had better control over their overall health (27/46), and had an overall pleasant experience (30/46). More than 50% (29/46) did not agree that using the smartwatch made them feel uncomfortable.

### Demographic Variables

We explored which demographic variables contributed to the usability (SUS), acceptance (TAM), and continuous use (EECM) of biosensors. We included age, level of education, and gender in separate 2-way ANCOVAs as the dependent variables were correlated, which prohibited a multifactorial multivariate analysis of covariance. In addition, we performed a Box-Cox transformation on the SUS and TAM as these were not normally distributed; the ANCOVAs were adjusted for age. There was a significant difference for both acceptance (F_1,69_=9.214, *P*=.003) and continuous use (F_1,69_=16.607, *P*<.001) for group; in both cases the clients scored higher on acceptance and intention for continuous use. There was no significant interaction between gender and education on the usability score (F_2,64_=0.549, *P*=.58), acceptance score (F_2,64_=0.545, *P*=.58), or continuous use score (F_2,64_=1.475, *P*=.24). This indicates that the effect of gender on usability, acceptance, and continuous use does not depend on the level of education or vice versa. As can be seen in Table 4, a strong correlation between the acceptance (TAM) of wearable devices and the intention of continuous use (EECM) was found. In addition, moderate correlations were found between usability with acceptance and continuous use. Last, there was a weak negative correlation between usability and age. Further analysis of the correlations showed that the staff members obtained strong correlations between usability and acceptance (*r*=.80, *P*<.001), usability and continuous use (*r*=.79, *P*<.001), and acceptance with continuous use (*r*=.89, *P*<.001). Clients obtained moderate correlations between usability and acceptance (*r*=.46, *P*=.01), usability and continuous use (*r*=.52, *P*=.003), and strong correlations between acceptance and continuous use (*r*=.75, *P*<.001).

**Table 4:** Spearman correlations between variables.

Questionnaire	SUS^a^	EECM^b^	TAM^c^
EECM	0.54	—^d^	—
TAM	0.58	0.86	—
Age	–0.24	–0.15	–0.03

^a^SUS: System Usability Scale.

^b^EECM: extended expectation confirmation model.

^c^TAM: Technology Acceptance Model questionnaire.

^d^Not applicable.

**Figure 7:**
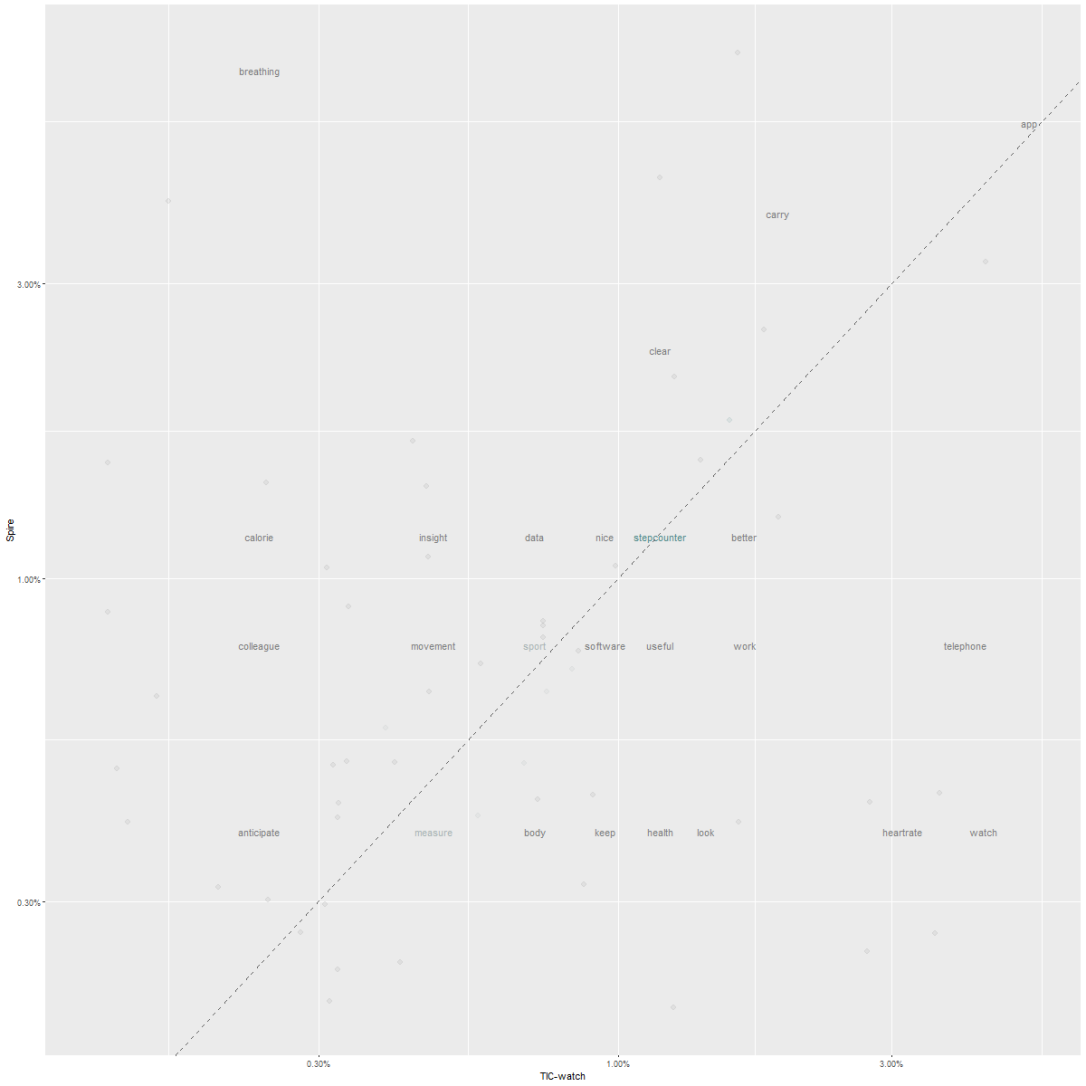
Word frequencies for the Spire Stone and TicWatch E.

For analysis of qualitative questionnaires, a sample size between 5 and 50 is required [30]. In our sample, only the qualitative interviews for the Spire Stone (n=6) and TicWatch E (n=10) met this criterion and are reported here. The word frequencies for both devices are reported in Figure 7 as we wanted to compare the frequencies for the different devices. Words that are close to the dotted line share a frequency similarity for both devices. Words that are farther away from the line have nonsimilar frequencies for both devices. As expected, breathing has a higher frequency for the Spire Stone and heart rate is higher for the TicWatch E as this is the main function for both devices. The Spire Stone and TicWatch E are generally considered easy to use and give clear information.

## Discussion

### Principal Findings

In this study, we investigated whether the expectancy or the actual experience was most important for an intended continuous use of biosensor devices for monitoring and coaching in forensic psychiatry. In addition, we investigated what contributes to the usability, acceptance, and intended continuous use. The main result of the study is that it was the actual experience of wearing a biosensor that was associated with intended continuous use, and to a much lesser extent, the expectancy. This is contrary to the hypotheses of Pal et al [12] that expectancy would be important for continuous use and that a gap exists between the expectations of usability and the factors that would lead to continuous use by experiences with the device. In our study, the expected usability had only a weak positive association with continuous use, while the actual experienced usability had a moderate positive association. These associations were markedly different between staff members and clients. The associations between usability and both acceptance and continuous use were stronger in staff members and more moderate in clients. It may well be that that factors outside usability are responsible for associations with acceptance and continuous use in clients. Perhaps the questionnaires on acceptance and continuous use do not cover the full range of aspects associated with usability for clients. It may also be that clients had different expectations and experiences with the device. Further longitudinal research (ie, longer than 1 month) on this association is warranted, and it would be of interest to investigate any mediation effects of technology experience on the expectancy–continuous use association to further investigate the hypothesis. This would, however, require larger sample sizes.

In addition, a strong association between the acceptance (TAM) of wearable devices and the intention of continuous use (EECM) was found. This seems to indicate that these two questionnaires measure overlapping constructs, and the question arises whether both need to be administered. Especially when the load on participants should be kept to a minimum as in our sample with forensic MID-BIF clients. These results must be interpreted with care as the design of our study, without proper counterbalancing, and the use of short questionnaires limit the conclusions that can be drawn from the study.

As far as the determinants for usability, acceptance, and continuous use are concerned, answers from the usability scale indicated that most of the clients and staff members felt confident using the biosensors and after they wore the devices and thought that most people would learn to use the product very quickly and want to use it frequently. The acceptance scale indicated that the majority have positive attitudes toward technology, their affective quality, relative advantage, mobility, availability, and perceived ease of use. The continuous use scale showed that the majority of staff members and clients gave positive answers on satisfaction, self-socio motivation, perceived comfort, and hedonic motivation. However, the majority had doubts on the perceived accuracy and functional limitations. It is also interesting to note that a minority of staff members and clients were not positive about the usability, acceptance, and continuous use of the devices. These people might not want to use the devices or think that they need help in using the devices. For instance, a minority of people think that they would need help from a technical person to use the device. It might well be that providing them with proper support might increase their intention to use the device. Also, some find that wearing the device is uncomfortable and the accuracy of the fitness data could be improved. These devices might thus benefit from developments in accuracy and form factor [2,3,15,31]. Also, it is unclear if questions on cost of the device can be properly answered as the participants in this study did not have to pay for the devices. Participants can only guess if the device was expensive. In addition, there were no differences in gender, education, or age for usability, acceptance, and continuous use. However, it must be pointed out that our sample size was limited. Clients showed a higher score on acceptance and intention for continuous use. It must be further investigated whether this is a true effect or could be due to a social desirability bias in the forensic clients. It is interesting that clients scored higher on acceptance and continuous use as they might benefit from the ease of use of these devices and their continuing monitoring and coaching apps.

### Strengths and Limitations

Two particular strengths of this study are the use of simply worded questionnaires adapted for clients with MID-BIF as this was not available in the literature. In addition, we used qualitative questionnaires and a diverse and heterogeneous sample as Kalantari [11] pointed out was missing in the literature. Three major limitations of this study are related to the validity of the questionnaires, design of the study, and duration of wearing the devices. First, although the reliabilities of the short TAM and EECM are above .80, it remains to be established if a short version of these questionnaires can validly capture the construct intended by the original questionnaires. Furthermore, it is unknown whether these short questionnaires measure similar constructs over time and for different groups. Second, the design of the study does not warrant any comparison between devices, as the participants did not wear all 4 devices. This seriously limits the comparability of results to a within-subject comparison on the SUS. Third, participants wore the devices for only 1 week, which will have a significant impact on the measure of continuous use. The measure only applies to expectations and intentions of continuous use. It remains to be established if this intention for continuous use is associated with actual continuous use. Further limitations of this study were the limited sample size, uneven number of administered questionnaires per device, and use of a modified version of the TAM. The original theoretical framework of the TAM [27] was altered in an extended smartwatch-oriented TAM [15] and it is unclear how this may have affected the results. Furthermore, one of the devices was not a smartwatch in the strict sense, and this limits the conclusions that can be drawn for this device.

### Future Research

Future research should focus on longitudinal research investigating usability, acceptance, and continuous use, should include a counterbalanced design in which all devices are worn at least once, and should investigate measurement invariance for the short questionnaires [32,33]. Biosensors in forensic psychiatry might prove to be a very useful addition in the treatment for vulnerable MID-BIF clients due to their health-related functionality. These clients often suffer from obesity, sleep disorders, and trauma-related disorders and might substantially benefit from the different functionality that biosensors potentially offer. The use of these devices yields high expectations, and it is plausible that wearable biosensor devices can be used to detect changing levels of emotional states, assist in self-regulation, and even signal imminent problem behavior, such as aggression in clients [14]. The types of biosensors that a client or staff member uses will depend, at least in part, on the problem (ie, health problems, sleep problems, fitness tracking) or use case (ie, emotion regulation, behavior modification) they have. Information on breathing and tension or focus during activities and at certain locations requires a different sensor than monitoring real-time heart rate changes, and the conditions in which the devices are used and prerequisites for using the biosensors should be as clear as possible to increase the use and maximize the potential health benefits of these sensors.

Another important topic for future research is the reliability and validity of the sensors, especially in comparison with gold standard equipment used in laboratories. Peake et al [11] reported that only 5% of wearable devices with integrated biosensors are well validated and most validation studies lack clear conclusions [34]. Not providing accurate and timely information might seriously affect the willingness to wear a device.

Last, for people with MID-BIF, it is especially important to develop easy-to-use biosensors with a minimum requirement on cognitive capacity to increase usability, acceptance, and continuous use in the future. It must be noted, however, that clients scored similar to staff members on ease of use of available devices and higher on acceptance and (intended) continuous use. Whether clients indeed grasped the information provided by the sensors must be investigated further.

### Conclusion

Actual perceived usability of wearing a biosensor and to a much lesser extent the expectancy of usability were associated with continuous use. Clients with mild intellectual disabilities might benefit from the ease of use of wearables devices and their continuing monitoring and coaching apps. Clients scored higher on acceptance and intention for continuous use, but associations between usability and both acceptance and continuous use were markedly stronger in staff members. For clients, it is especially important to develop easy-to-use biosensors with a minimum requirement on cognitive capacity to increase usability, acceptance, and continuous use.

## Appendix

Multimedia Appendix 1 Short questionnaires used in the study.

## Abbreviations

ANCOVA: analysis of covariance
EECM: extended expectation confirmation model
MID-BIF: mild intellectual disabilities and borderline intellectual functioning
SUS: System Usability Scale
TAM: Technology Acceptance Model questionnaire
