# Supplementary material for: Usability and Acceptance of Wearable Biosensors in Forensic Psychiatry: Cross-sectional Questionnaire Study
Source: JMIR Form Res. 2021 May 10;5(5):e18096. doi: 10.2196/18096 (PMC8145084; doi:10.2196/18096)
Supplement: Multimedia Appendix 1 [file formative_v5i5e18096_app1.pdf]

Wilt u zo vriendelijk zijn om het juiste antwoord te omcirkelen of in te vullen?

Onderzoeksnummer :

Datum:

1. Welk product heeft u gedragen?	Fitbit	Garmin	Spire	Ticwatch

2. Wat is uw geslacht?	Man	Vrouw

3. Wat is uw leeftijd	

4. Wat is uw hoogst genoten opleiding waarvoor u een diploma hebt behaald?	Lagere school	VMBO/MAVO	HAVO	VWO	MBO	HBO	Universiteit

5. Over het algemeen zou ik de gebruiksvriendelijkheid van dit product beoordelen als …	

	Helemaal mee eens	Mee eens	Weet niet, neutraal	Mee oneens	Helemaal oneens
SUS1: Ik wil het product vaker gebruiken.	5	4	3	2	1
SUS2: Ik vind het product ingewikkeld.	5	4	3	2	1
SUS3: Het product is moeilijker te gebruiken dan ik dacht.	5	4	3	2	1
SUS4: Ik denk dat ik hulp moet hebben om het product te gebruiken.	5	4	3	2	1
SUS5: Ik vond dat het apparaatje en de app goed bij elkaar passen.	5	4	3	2	1
SUS6: Ik vind dat het apparaatje en de app niet bij elkaar passen.	5	4	3	2	1
SUS7: Ik denk dat veel mensen het product heel snel leren gebruiken.	5	4	3	2	1
SUS8: Ik vond het product erg moeilijk om te gebruiken.	5	4	3	2	1
SUS9: Ik denk dat ik om kan gaan met het product.	5	4	3	2	1
SUS10: Ik moest veel leren over het product voordat ik het kon gaan gebruiken.	5	4	3	2	1

	Helemaal mee eens	Mee eens	Beetje mee eens	Weet niet, neutraal	Beetje mee oneens	Mee oneens	Helemaal oneens
TAMAT3: Het bevalt mij goed om dit product te gebruiken.	7	6	5	4	3	2	1
TAMPE2: Ik vind het makkelijk om dit product te gebruiken.	7	6	5	4	3	2	1

	Helemaal mee eens	Mee eens	Beetje mee eens	Weet niet, neutraal	Beetje mee oneens	Mee oneens	Helemaal oneens
TAMPU3: Dit product kan ik goed gebruiken bij de dingen die ik moet doen.	7	6	5	4	3	2	1
TAMAQ3: Dit product ziet er mooi uit en ik heb er plezier van.	7	6	5	4	3	2	1
TAMRA2: Dit product heeft meer goede kanten dan slechte kanten.	7	6	5	4	3	2	1
TAMMB2: Dit product kan ik overal gebruiken.	7	6	5	4	3	2	1
TAMAV2: Dit product geeft mij informatie wanneer ik het wil.	7	6	5	4	3	2	1
TAMSA1: Dit product maakt dat ik anders ben dan andere mensen.	7	6	5	4	3	2	1
TAMCT1: Ik denk dat het product duur is.	7	6	5	4	3	2	1
EECMPU3: Dit product maakt veel dingen makkelijker.	7	6	5	4	3	2	1
EECMST2: Ik vond het fijn dit product te gebruiken.	7	6	5	4	3	2	1
EECMCF1: Dit product was beter dan ik had verwacht.	7	6	5	4	3	2	1
EECMCF3: Dit product kan meer dan ik dacht.	7	6	5	4	3	2	1
EECMHM3: Ik heb plezier als ik dit product gebruik.	7	6	5	4	3	2	1
EECMSsM2: Mensen die belangrijk voor me zijn vinden het een goed idee dat ik dit product gebruik.	7	6	5	4	3	2	1
EECMSsM4: Door dit product word ik gezonder.	7	6	5	4	3	2	1
EECMPP3: Andere mensen kunnen de informatie van dit product stelen.	7	6	5	4	3	2	1
EECMPC1: Ik kan dit product niet te lang gebruiken omdat hij zwaar en groot is.	7	6	5	4	3	2	1
EECMPC2: Soms voel ik me niet fijn als ik dit product gebruik.	7	6	5	4	3	2	1
EECMBlC2: De batterij van het product gaat niet lang mee.	7	6	5	4	3	2	1
EECMPAFL1: Ik denk dat de informatie van het product klopt.	7	6	5	4	3	2	1
EECMCU1 en TAMIU2: Ik wil dit product blijven gebruiken.	7	6	5	4	3	2	1
